# Identifying genome-wide immune gene variation underlying infectious disease in wildlife populations – a next generation sequencing approach in the gopher tortoise

**DOI:** 10.1186/s12864-018-4452-0

**Published:** 2018-01-19

**Authors:** Jean P. Elbers, Mary B. Brown, Sabrina S. Taylor

**Affiliations:** 1 School of Renewable Natural Resources, 227 RNR Bldg., Louisiana State University and AgCenter, Baton Rouge, LA 70803 USA; 2 Department of Infectious Diseases and Pathology, College of Veterinary Medicine, University of Florida, Gainesville, FL 32611 USA

**Keywords:** Genome-wide association study, Target enrichment, Sequence capture, Next-generation sequencing, Immunogenetics, *Gopherus polyphemus*, Immunomes

## Abstract

**Background:**

Infectious disease is the single greatest threat to taxa such as amphibians (chytrid fungus), bats (white nose syndrome), Tasmanian devils (devil facial tumor disease), and black-footed ferrets (canine distemper virus, plague). Although understanding the genetic basis to disease susceptibility is important for the long-term persistence of these groups, most research has been limited to major-histocompatibility and Toll-like receptor genes. To better understand the genetic basis of infectious disease susceptibility in a species of conservation concern, we sequenced all known/predicted immune response genes (i.e., the immunomes) in 16 Florida gopher tortoises, *Gopherus polyphemus.* All tortoises produced antibodies against *Mycoplasma agassizii* (an etiologic agent of infectious upper respiratory tract disease; URTD) and, at the time of sampling, either had (*n* = 10) or lacked (*n* = 6) clinical signs.

**Results:**

We found several variants associated with URTD clinical status in complement and lectin genes, which may play a role in *Mycoplasma* immunity. Thirty-five genes deviated from neutrality according to Tajima’s *D*. These genes were enriched in functions relating to macromolecule and protein modifications, which are vital to immune system functioning.

**Conclusions:**

These results are suggestive of genetic differences that might contribute to disease severity, a finding that is consistent with other mycoplasmal diseases. This has implications for management because tortoises across their range may possess genetic variation associated with a more severe response to URTD. More generally: 1) this approach demonstrates that a broader consideration of immune genes is better able to identify important variants, and; 2) this data pipeline can be adopted to identify alleles associated with disease susceptibility or resistance in other taxa, and therefore provide information on a population’s risk of succumbing to disease, inform translocations to increase genetic variation for disease resistance, and help to identify potential treatments.

**Electronic supplementary material:**

The online version of this article (10.1186/s12864-018-4452-0) contains supplementary material, which is available to authorized users.

## Background

Several species of conservation concern are in direct danger of extinction from disease. For example, devil facial tumor disease, a contagious cancer, is driving Tasmanian devils (*Sarcophilus harrisii*) to extinction at an alarming rate [1]. Similarly, the last native black-footed ferret (*Mustela nigripes*) population crashed as a result of canine distemper disease. Disease and reintroduced populations are susceptible to plague [2]. Further millions of bats and amphibians have died as a result of white-nose syndrome and chytrid fungus, respectively [3, 4].

Some diseases are projected to spread as a result of changing land use and climate as well as through anthropogenic spread [5]. For example, ticks (*Ixodes scapularis*), and the associated pathogens they vector, are expected to spread north as a consequence of climate change [6]. Other examples of diseases predicted to spread include West Nile virus, sarcoptic mange, and cholera ([7–9], see [10] for review). Regardless of the driver, infectious diseases in plant and animal populations are spreading and threatening many species of conservation concern.

Infectious disease susceptibility in plants and animals may have a genetic basis, as changes in the coding portions of immune response genes can affect protein conformation and alter pathogen recognition and binding [11]. Most studies have focused on a handful of immune genes (e.g. MHC (major histocompatibility complex), TLR (Toll-like receptors)) although several hundred genes are involved in the immune response. Genetic variants underlying disease susceptibility throughout the genome may be identified by associating immunogenetic variation and disease clinical status in a genome-wide association study (GWAS). GWASs are commonly conducted with agricultural crops (e.g., [12]), model organisms (e.g., [13]), and humans (e.g., [14]) to look for genetic variation associated with disease susceptibility. For example, researchers found 18 candidate genes associated with resistance to head smut disease in corn, leading to improved corn cultivar selection in an important agricultural crop [12]. Similarly, GWAS work in humans has helped identify the mechanisms of hepatitis C infection [14]. In contrast, GWAS in wildlife populations has been very limited despite its tremendous potential to identify the genetic basis of disease resistance and susceptibility [15–20]. For example, investigators used restriction-site associated DNA sequencing (RADseq) to find associations between candidate loci and bacterial cold water disease resistance in rainbow and steelhead trout [17]. Seven single nucleotide polymorphisms (SNPs) were associated with survival status and/or time, which could presumably be used to increase fitness in aquaculture or wild stocks.

The gopher tortoise, *Gopherus polyphemus*, is a threatened tortoise that can contract a chronic, and occasionally fatal, infectious upper respiratory tract disease (URTD) caused by *Mycoplasma agassizii* [21]. There may be a genetic component to URTD susceptibility as mycoplasmas are widespread, but gopher tortoise die-offs attributable to URTD have been documented only in Georgia and Florida [22, 23]. Tortoise populations show genetic structure at microsatellite loci between URTD-positive and -negative regions, thus there may also be genetic differences in immune response genes [24].

The gopher tortoise is federally listed as threatened in the portion of its range west of the Mobile and Tombigbee Rivers in Alabama, and the eastern tortoises are candidates for listing on the Endangered Species Act [25]. As such, the tortoise has been the subject of many genetic studies using neutral genetic markers such as microsatellites to inform management (e.g., [24]), but no research has identified genetic variation associated with susceptibility or resistance to URTD. This is crucial because tortoise populations may have adequate levels of genetic variation based on neutral microsatellites, but may be depauperate in adaptive genetic variation that influences immune responses to infections such as URTD, and therefore, population viability. Herein, we identify synonymous and non-synonymous variants in hundreds of genes that influence immune responses. We examined which variants associate with URTD non-clinical and clinical gopher tortoises to better understand the genetic basis for susceptibility to URTD. We also evaluate population genetic diversity, differentiation, and admixture among individuals from three populations in Florida. Finally, we assess which SNPs may be under selection, which genes may deviate from neutrality, and the specific functions of these non-neutral loci. We undertake this work using a data pipeline that can be used to identify important immune gene variants in a wide range of wildlife species in danger of extinction from disease.

## Methods

### Samples

**Fig. 1:**
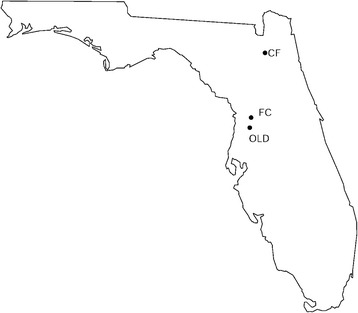
Location of Florida study populations. CF for Cecil Field/Branan Field Wildlife and Environmental Area, FC for Fort Cooper State Park, and OLD for Perry Oldenburg Wildlife and Environmental Area

Between 2002 and 2006, 11 populations in Florida were sampled annually as part of a long-term field study of gopher tortoise health [26]. Serum and erythrocytes were collected from all animals, and red blood cells were stored at − 80 °C until deoxyribonucleic acid (DNA) extraction. We selected 16 gopher tortoises representing two clinical states (tortoises with and without clinical signs of URTD; Table 1) from 3 populations (Cecil Field/Branan Field Wildlife and Environmental Area (CF), Fort Cooper State Park (FC), and Perry Oldenburg Wildlife and Environmental Area (OLD); Fig. 1) that had documented URTD. The sites chosen represented populations at different transition stages of URTD. The FC site has been studied since 2003, and from 2003 to 2005, seroprevalence to *M. agassizii* was extremely low, and mortality events and observed clinical disease were rare. However, in 2006, increases in seroprevalence, clinical signs, and mortality events were observed, indicating that the FC site had transitioned from an *M. agassizii* URTD-negative site to an acute epizootic site. Prior to 2003, the seroprevalence of *M. agassizii* at the CF site ranged from 0 to 20%. In 2003, dramatic increases were seen in seroprevalence (77% positive), tortoises exhibited a nasal discharge (33%), and tortoises (58%) positive by PCR for *M. agassizii*. Mortality events were observed, seroprevalence remained high, but clinical disease expression and severity decreased over time, suggesting that the CF site had transitioned from an acute epizootic site to an endemically stable site. The OLD site was endemically stable but had experienced a large-scale die-off circa 1996–1998 attributed to URTD [26]. In 2006, a sharp increase in seroconversion was observed, with an increase in clinical signs and slight increases in mortality events, supporting the concept that the OLD population was undergoing recrudescence of mycoplasmal URTD. All 16 tortoises in the 2 clinical states were observed more than once between 2002 and 2006 and had a least one positive enzyme-linked immunosorbent assay indicating active production of antibodies against *M. agassizii* [27]. Clinical tortoises had at least one incident of mild to severe nasal discharge. Because tortoises with URTD have intermittent expression of clinical signs, an important caveat is that tortoises were observed only once for several hours each year at time of capture, and therefore we cannot rule out the possibility that animals without clinical signs had previously been clinically ill. In experimental infections an individual can show clinical signs one day, be subclinical, and then present again with clinical signs [21]. However, at each time of capture, non-clinical tortoises were never observed with nasal discharge, an important diagnostic clinical sign of URTD.

**Table 1:** Description of *Gopherus polyphemus* samples by clinical status and diploid genotypes at several bases along the gene LOC101950941 on NCBI contig NW_007281632.1

Sample	Clinical status	NW_007281632.1 Bases				
		53,575	53,576	53,577	53,578	53,579
FC19	Non-clin	A,A	A,A	T,T	A,A	AG,AG
OLD77	Non-clin	A,A	A,A	T,T	A,G	AG,AG
CF53	Non-clin	A,A	A,A	T,T	A,A	AG,AG
CF69	Non-clin	A,A	AT,AT	G,G	A,A	A,A
FC58	Non-clin	A,A	A,AT	G,T	A,G	A,AG
OLD106	Non-clin	A,A	AT,AT	G,G	A,A	A,A
CF72	Clin	A,A	AT,AT	G,T	A,A	A,A
CF80	Clin	A,A	AT,AT	G,G	A,A	A,A
CF90	Clin	A,A	AT,AT	G,G	A,A	A,A
CF219	Clin	A,A	AT,AT	G,G	A,A	A,A
FC13	Clin	A,A	AT,AT	G,G	A,A	A,A
FC15	Clin	A,A	AT,AT	G,G	A,A	A,A
FC47	Clin	A,A	AT,AT	G,G	A,A	A,A
OLD65	Clin	A,A	AT,AT	G,G	A,A	A,A
OLD92	Clin	A,A	AT,AT	G,G	A,A	A,A
OLD107	Clin	A,A	AT,AT	G,G	A,A	A,A

Non-clin for tortoises that were never observed with nasal discharge during the duration of the field study and Clin for tortoises that had at least one incident of mild to severe nasal discharge

*CF* Cecil Field, *FC* Fort Cooper, and *OLD* Oldenburg

### Target enrichment

We used a next-generation sequencing approach and previously developed ribonucleic acid (RNA) baits [28, 29] from MYcroarray Inc. (Ann Arbor, MI, USA) in an in-solution hybridization experiment to capture the immunomes of 16 gopher tortoises. Briefly, we used the GO2TR workflow [24] to design a target region of exons from *Chrysemys picta bellii* (western painted turtle) genome [25] relating to the gene ontology term “immune response” to create 120-bp RNA baits. We followed the steps of a previous immunome study [28], except we created libraries using Bioo Scientific Nextflex Pre-and Post-Capture Combo Kit-Set A (catalog no. 5144–51, Bioo Scientific Corp., Austin, TX, USA) and used Bioo Scientific reagents and protocols for target enrichment.

### Data analysis

We used the same analysis pipeline of [28] to call SNPs and indels and used those previously identified variants for variant quality score filtering. Briefly, we performed adapter and quality trimming with TRIMMOMATIC v0.32 [30], merged overlapping paired-end reads with BBMerge v5.4 (https://sourceforge.net/projects/bbmap/), aligned merged and unpaired reads and paired reads separately to the *C. p. bellii* 3.0.3 genome [31] using BWA-MEM v0.7.12 [32], then less stringently with STAMPY v1.0.23 [33], and followed the Genome Analysis Toolkit’s v3.3.0 [34] best practices for variant calling using the Haplotype Caller. For population genomic analyses, we used the same programs and steps as [28] except we included di- (*n* = 16,169), tri- (*n* = 651), and tetra-allelic (*n* = 1) SNP loci. To determine what polymorphic variants were under selection, we used BayeScan v2.1 [35], an *F*_*ST*_ outlier test, and used a false discovery rate [36] of 0.1.

We calculated Tajima’s *D* [37] for each population separately using VariScan [38] for all gene regions (i.e., loci) that were at least 60 bp and had reads at ≥20X coverage. We used a variable cutoff for significant negative and positive values of Tajima’s *D* based on the empirical *P* value calculated for each population and locus combination as follows: first we simulated extreme values of Tajima’s *D* using 1000 simulations for a population of five individuals with a single locus containing 1–138 segregating sites using the program ms [39]. Then we used custom R [40] code to calculate the *P* value of the empirical Tajima’s *D* given the simulated extreme values for a particular number of segregating sites and retained only loci with a false discovery rate less than 0.05. We performed functional enrichment analysis using FatiGO [41] implemented in Blast2GO v3.1 [42] to determine if non-neutral genes were enriched for specific biological functions relative to rest of genome.

We labeled alternate SNPs and indels as synonymous or non-synonymous using snpEff v4.0e [43]. We performed case/control association tests with PLINK v1.07 [44], analyzing SNPs and indels separately. We repeated association test using ROADTRIPS v2.0 [45], which controls for unknown population structure and individual relatedness.

## Results

From two Illumina MiSeq runs using 75-bp paired end reads, we obtained 21.5 million reads that passed quality control, and each tortoise had 1.3 ± 0.2 (mean ± SD) million reads. Mean coverage was 47.5 ± 7.6 reads (Additional file 1: Figure S1 and Additional file 2: Figure S2). We targeted 632 immune genes and 5425 exons, and each tortoise had reads for 593.9 ± 3.5 genes and 4198 ± 82.3 exons. After variant filtering, imputation, and removing non-polymorphic loci, we identified 16,821 SNPs and 3226 indels.

SNP allelic richness was lowest in CF, intermediate in OLD, and highest in FC. Observed and expected heterozygosity was lowest in CF but equivalent in FC and OLD (Table 2). Pairwise *F*_*ST*_ values were 0.027 (CFxFC), 0.060 (CFxOLD), and − 0.003 (FCxOLD): low values were expected based on geographic proximities of the populations.

**Table 2:** Estimates of genetic diversity for each tortoise population

Site	AR	HO	HE
CF	1.75^a^	0.32^a^	0.29^a^
FC	1.78^c^	0.33^b^	0.30^b^
OLD	1.77^b^	0.34^b^	0.30^b^

*AR* allelic richness, *HO and HE* for observed and expected heterozygosity, respectively, CF Cecil Field, *FC* Fort Cooper, and *OLD* Oldenburg

Superscript letters for each column indicate differences at the 0.05 level using a post-hoc Tukey test. Shared letters within a column indicate no difference while unique letters indicate differences in levels

**Fig. 2:**
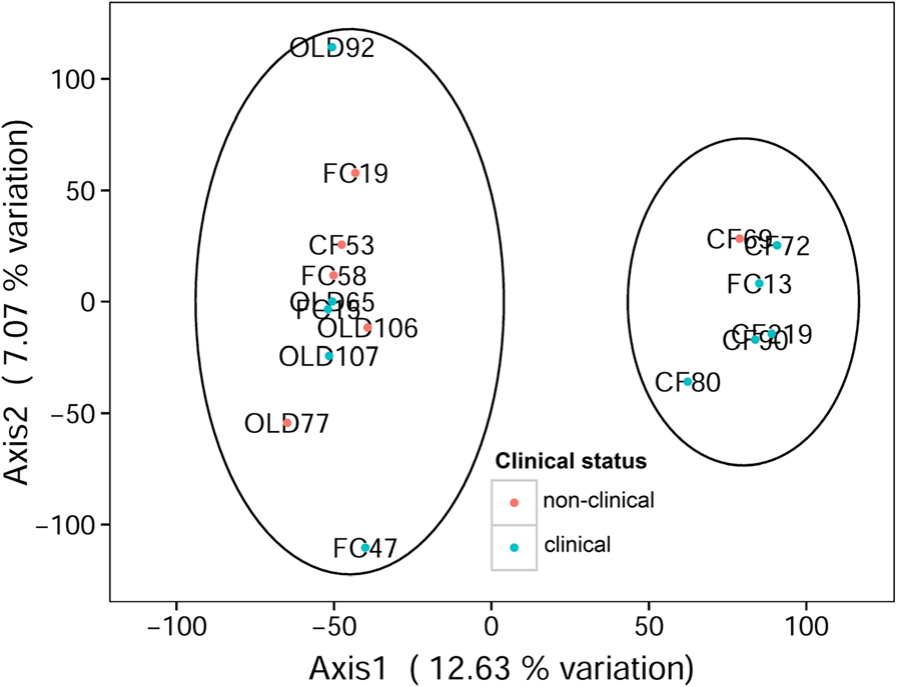
Principle component analysis for 16,821 all polymorphic SNPs. Circles indicate optimum clusters identified using STRUCTURE and STRUCTURE HARVESTER

**Fig. 3:**
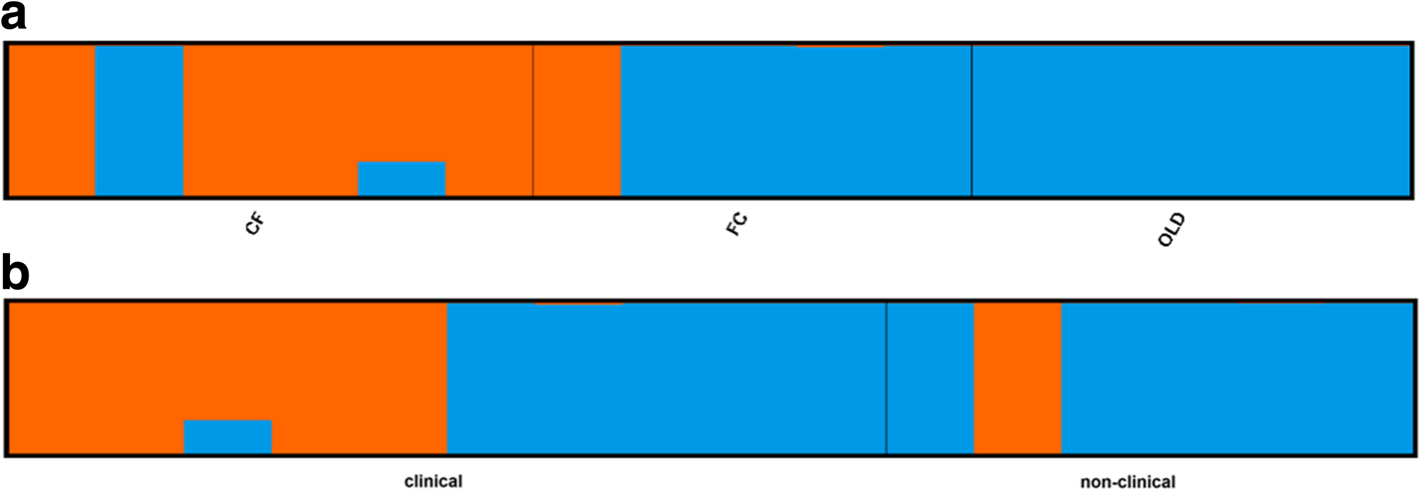
STRUCTURE plots using (**a**) populations and (**b**) clinical status with optimum number of clusters *K* = 2 determined by STRUCTURE HARVESTER. CF for Cecil Field/Branan Field Wildlife and Environmental Area, FC for Fort Cooper State Park, and OLD for Perry Oldenburg Wildlife and Environmental Area. Non-clinical tortoises were never observed with nasal discharge during the duration of the field study. Clinical tortoises had at least one incident of mild to severe nasal discharge

Population admixture inferred with SNPs using principle component analysis (PCA, Fig. 2) and STRUCTURE ([46], Fig. 3a) suggested two clusters: the first with CF and the second with FC and OLD mixed together. These results agreed with the optimal *K* value of two based on STRUCTURE HARVESTER [47]. There was no meaningful grouping of samples when we used clinical status to cluster individuals in STRUCTURE (Fig. 3b).

There were two SNPs but no indel loci putatively under selection. The first SNP, NW_007281406.1:2,298,828 was non-synonymous and present in the 1st codon position of the 354th amino acid in the gene IFIT1-like. The second SNP, NW_007322484.1:105, was synonymous and found in the gene IFI44-like.

There were 1556 polymorphic gene regions of which 70 deviated from neutrality. Tajima’s *D* was negative (i.e., ≤ − 1.56) for 27 regions suggesting either population expansion or positive selection but positive (≥ 2.06) for 43 regions suggesting either population bottlenecks, balancing selection, or strong population structure. These regions represented 35 genes (Additional file 3: Table S1). Thirteen genes had positive, 19 genes had negative, and 3 genes (IPO11, SIN3A, and TRAF6) had both positive and negative (i.e., multiple regions occurred in the same gene) Tajima’s *D* values.

**Fig. 4:**
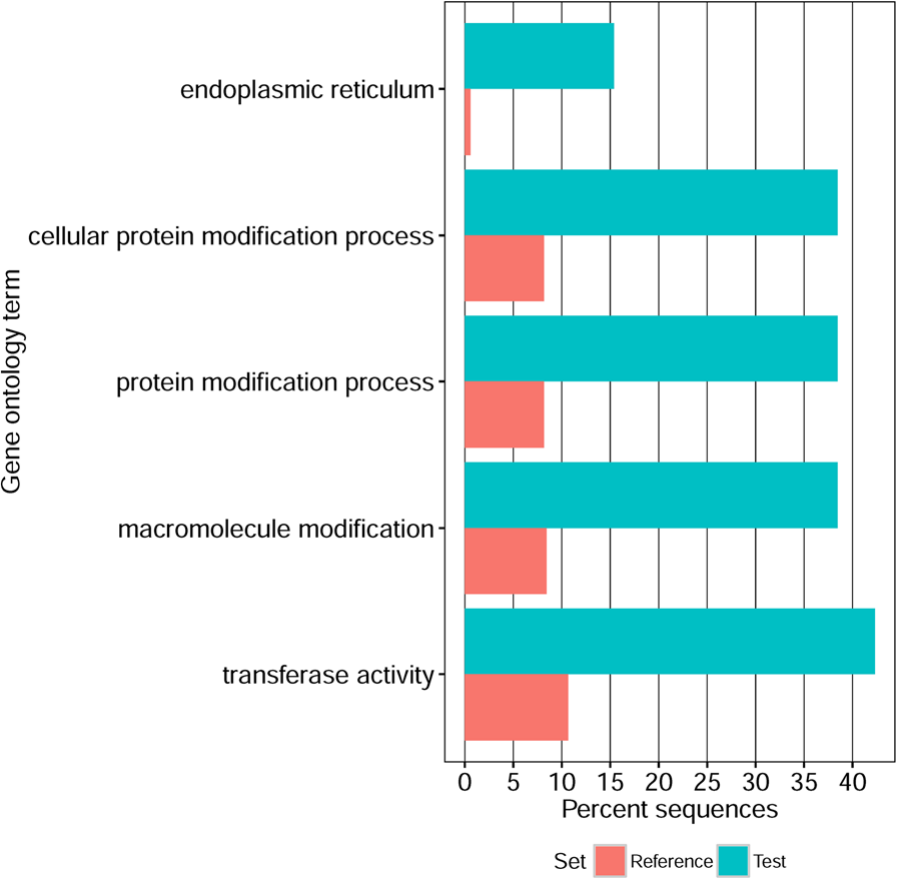
Functional enrichment analysis of gopher tortoise genes deviating from neutrality (i.e., Test) compared to the rest of the genome (i.e., Reference) based on percent of sequences with particular gene ontology terms

Functional enrichment analysis of the 35 genes that contained regions deviating from neutrality showed overrepresentation of several gene functions, most notably the gene ontology terms: endoplasmic reticulum, cellular protein modification process, protein modification process, macromolecule modification, and transferase activity (Fig. 4).

Association tests with PLINK revealed several SNPs and indels (Table 3, Additional file 4: Table S2) that were associated with clinical status but which were not significantly associated after correction for multiple tests. Variants that lack statistical significance are typical of GWAS studies, especially when variant effect sizes are small, unless hundreds or thousands of subjects are examined [48], therefore it is important to use less strict significance criteria and report the top ranking variants (e.g., [12]). Association tests with ROADTRIPS produced the same list of SNPs and indels as PLINK (data not shown). SNPs with the strongest associations were contained in genes such as *Austrelaps superbus* venom factor 1-like and LOC101950941. Indels with the strongest associations were in genes such as LOC101950941 and TNFRSF5-like.

**Table 3:** Top SNPs and indels from PLINK GWAS association analysis between affected (clinical) and unaffected (non-clinical) tortoises

Position	Gene	Type	Unadjusted *P*
SNPs^a^			
NC_024232.1:1,105,959	A. superbus venom factor 1-like	non-syn	0.0001388
NC_024232.1:1,105,940	A. superbus venom factor 1-like	syn	0.0001388
NC_024232.1:1,105,284	A. superbus venom factor 1-like	syn	0.0001388
NW_007281632.1:53,578	Uncharacterized LOC101950941	non-syn	0.0001725
NW_007281632.1:53,577	Uncharacterized LOC101950941	non-syn	0.0001725
Indels^b^			
NW_007281632.1:53,576	Uncharacterized LOC101950941	syn	0.0001725
NW_007281632.1:53,579	Uncharacterized LOC101950941	non-syn	0.0001725
NC_024234.1:2,112,869	TNFRSF5-like^c^	syn	0.0007433

*Syn* synonymous and *non-syn* non-synonymous

^a^All of these SNPs had a false discovery rate of 0.4164 after correction for multiple tests

^b^All of these indels had a false discovery rate of 0.2193 after correction for multiple tests

^c^Tumor necrosis factor receptor superfamily member 5-like

We found alleles at two non-synonymous SNPs and one non-synonymous indel shared by all but one URTD clinical tortoise (CF72, observed twice, once with URTD signs and again without) but also shared by two non-clinical animals (CF69 and OLD77). The SNPs were found on the following National Center for Biotechnology Information (NCBI) contig NW_007281632.1 at positions 53,577 and 53,578, and the indel was found at position 53,579 (Table 1). All were located in the gene LOC101950941.

## Discussion

We sequenced the immunomes of 10 tortoises with clinical signs of URTD and 6 tortoises without clinical signs of URTD to: 1) estimate genetic diversity, differentiation, and admixture in each sampling location, and; 2) to identify SNPs putatively under selection, genes deviating from neutrality, and to better understand the genetic basis of URTD susceptibility, and; 3) to outline an approach that could be used to identify important genetic variants associated with diseases in other threatened species. We discuss each of these below.

Genetic differentiation and admixture were in line with expectations. First, pairwise *F*_*ST*_ values showed genetic differentiation corresponded to geographic distance. Second, genetic admixture inferred by PCA and STRUCTURE methods was congruent and reasonable as FC and OLD are geographically close and cluster together whereas CF is farther away and clusters separately. Because the populations represented three transition states in URTD epidemiology, differences also could be reflective of changes in the population after transition from initial URTD acute outbreaks to endemically stable and/or recrudescent outbreaks rather than geography alone. Clinically ill tortoises at CF could represent a subpopulation of more susceptible animals, which might be predicted to be among those impacted most during an initial disease outbreak. The historic OLD die-off [26] could have resulted in loss of the most susceptible animals from the population during the acute disease outbreak. Long term surveillance of populations would be needed to address this possibility.

Two SNPs appeared to be under selection. The first SNP, NW_007281406.1:2,298,828, was in the gene IFIT1-like; IFIT1 can bind to viral RNA with a triphosphate group on the 5′ end (i.e., PPP-RNA producing viruses, [49]). Specifically, the absence of IFIT1 leads to increased growth and pathogenicity of PPP-RNA producing viruses. The second SNP, NW_007322484.1:105, was synonymous and found in the gene IFI44-like; IFI44 may function in reducing viral transcription by suppressing long terminal repeat promoter activity of viruses such as HIV-1 [50]. Both of these SNPs are involved with response to viruses, and there are many viruses that affect turtles and tortoises (reviewed in [51]). For example, the genus *Ranavirus* as well as Herpesviruses affect *G. polyphemus* [52]. In fact, the *G. polyphemus* with *Ranavirus* examined by AJ Johnson, AP Pessier, JFX Wellehan, A Childress, TM Norton, NL Stedman, DC Bloom, W Belzer, VR Titus, R Wagner, et al. [52] was found only 50 km from the CF population. It is not clear if immunity to Ranaviruses or other viruses is influenced by variation in IFIT1 and IFI44.

There were 27 gene regions that had negative Tajima’s *D* values. We do not think this was due to population expansion, as *G. polyphemus* populations have declined across their range, especially during the past century [53]. Therefore, it is more likely that these genes are experiencing the effects of positive selection [54]. Forty-three regions had positive Tajima’s *D* values, which is unlikely to be due to population bottlenecks as estimates of genetic diversity are very high for the populations assessed in this study relative to other gopher tortoise populations [28]. These positive values may be due to balancing selection as we did not detect strong population structure between OLD and FC samples [54]. It is interesting that three genes (IPO11, SIN3A, and TRAF6) had regions that were potentially under positive and balancing selection. This finding may be because we only analyzed gene regions that were at least 60 bp and had 20× coverage resulting in partial gene sequences as opposed to the full gene sequences analyzed by other studies (e.g., [55]).

Functional enrichment analysis of the 35 gene regions that deviated from neutrality (Additional file 3: Table S1) suggested several things. First, these genes were enriched relative to the rest of the genome for gene ontology functions relating to the modification of macromolecules and proteins, which usually fold and mature in the lumen of the endoplasmic reticulum [56]. Second, these genes were enriched with transferase activity, which includes glycosylation of glycoproteins (i.e., adding sugars to glycoproteins) that can take place after glycoproteins leave the endoplasmic reticulum and travel to the Golgi apparatus (reviewed in [57]). Third, both macromolecule and protein modifications can also take place in Golgi apparatus, especially modifications for proteins destined to be secreted outside of cells [57], such as cytokines, which are vital to immune system functioning [58].

Genes that contained SNPs and indels with the strongest associations to clinical status may influence key biological functions in *Mycoplasma* immunity and are useful as hypotheses to test whether specific alleles may make tortoises more likely to present URTD signs. These variants were found in the *A. superbus* venom factor 1-like and LOC101950941 genes. *A. superbus* venom factor 1 (and possibly *A. superbus* venom factor 1-like) encodes a protein component of *Austrelaps superbus* (lowland copperhead snake) venom that is structurally and functionally similar to the complement protein C3 [59], which initiates the alternative pathway of the complement cascade, an important innate immune system process that results in the opsonization or lysis of pathogens and initiation of inflammatory responses [58]. *A. superbus* venom factor 1 may function like its analog C3 in mycoplasmal infections. Specifically, mycoplasmas such as *M. pulmonis* activate C3 [60], which can be broken down into C3a and C3b. C3b coats the surfaces of microbes, leading to opsonization or lysis [58].

LOC101950941 likely encodes a C-type lectin-like receptor found on natural killer cells (NK) based on a Coding Domain Search [61]. The full protein is predicted to be 405 amino acids in length, and the C-type lectin-like receptor domains are found between amino acids 70–154 and 283–400. The region between amino acids 70–154 contains the NW_007281632.1:53,577 and NW_007281632.1:53,578 SNPs and the NW_007281632.1:53,576 and NW_007281632.1:53,579 indels identified as important in this study. Because these SNPs and indels are non-synonymous (except NW_007281632.1:53,576), they may influence the functionality of the lectin receptors. Natural killer cells (NK) are part of the innate branch of the immune system and can detect unusual carbohydrates using lectin receptors to probe host cells for foreign or naturally occurring carbohydrates [58]. NK have a complicated role in mycoplasma immunity. First, NK are attracted to the site of mycoplasma infection in response to cytokines secreted by macrophages. Then NK produce INF-γ, an important cytokine that activates macrophages, in response to mycoplasma infection [62]. Interestingly, NK can be detrimental to clearing mycoplasma by negatively affecting the innate immune response [62]. In particular, mice depleted of NK and then infected with *M. pulmonis* have lower mycoplasmal load than mice with intact NK [63]. Thus, NK can have a negative influence on clearing of mycoplasmas by innate immune responses.

The strongest associated indels were present in the genes LOC101950941 and TNFRSF5-like. The indel at NW_007281632.1:53,579, which was contained within LOC101950941 was also found in our analysis of indels with non-synonymous alleles shared by tortoises with clinical signs. The TNFRSF5 gene (also referred to as CD40) encodes a receptor on antigen-presenting cells of the immune system such as dendritic and B cells as well as macrophages. TNFRSF5 functions in a variety of immune and inflammatory responses including the survival and proliferation of B cells [64] and induces the production of cytokines in dendritic cells and macrophages [58]. *Mycoplasma arthritidis* and *M. fermentans* have membrane-associated lipoproteins that upregulate CD40 expression by dendritic cells and macrophages [65–67], and it is likely that surface lipoproteins of *M. agassizii* could similarly impact CD40 upregulation. Therefore it is reasonable to suggest that there may be interactions between variation at the TNFRSF5-like gene and mycoplasma immunity in *G. polyphemus*.

There are several conservation implications based on the associations found between genetic variation and URTD clinical status. First, previous immunome work by JP Elbers, RW Clostio and SS Taylor [28] showed that 16 tortoises with unknown URTD clinical status from Louisiana, Alabama, Georgia, and Florida (4 per population) possessed the same alleles at SNPs NW_007281632.1:53,578 and NW_007281632.1:53,577 as the URTD clinical tortoises sequenced in this work. If the strong associations observed between genetic variation and URTD clinical status carry over to other tortoises, and these associations prove to be causal rather than correlated, then it is possible that tortoises across the range of the species possess genetic variation that makes them more likely to present URTD clinical signs. In particular, the Georgia population surveyed by JP Elbers, RW Clostio and SS Taylor [28] had the same variants as the URTD clinical tortoises and evidence of Mycoplasmal-URTD. Most tortoises from Green Grove, an area of Jones Ecological Research Center in Southwest Georgia, had been exposed to URTD since 125 out of 136 (92%) produced antibodies against *M. agassizii*; 44 out of 191 (23%) presented mild to severe clinical signs of URTD; and 101 out of 366 (28%) had evidence of chronic URTD lesions [22]. This suggests some correlation between the genetic variation associated with disease susceptibility and actual presentation of disease. If further experimental studies and more careful examination of these genes show a causal relationship, managers may wish to translocate tortoises from appropriate donor to at risk populations to bolster genetic variation at these loci. Future work might also examine the severity of Mycoplasmal-URTD infections, which may be influenced by particular immunome variants. In particular, some gopher tortoises may be susceptible to Mycoplasmal-URTD but possess immunome variants such that clinical signs are not severe, resulting in little to no impediment to normal function.

More generally, understanding the genetic basis of disease susceptibility is critical for a variety of reasons. First it can provide information on populations’ risk to succumbing to disease. For example, mice with the H-2D^k^ allele can properly recognize cytomegalovirus-infected cells [68], thus mice populations with low frequencies of this allele may be at increased risk of developing chronic infections, especially in the salivary glands. Second, identifying genetic mechanisms of disease resilience can inform management, in particular if managers are able to spread “beneficial” variation to populations that are depauperate in genetic variation at susceptibility loci by translocating genetically variable individuals. Finally, genetic understanding of disease predisposition can lead to potential treatments. For example, tumor cells in devil facial tumor disease (DFTD) are not recognized by the adaptive immune system of Tasmanian devils because the tumors do not express major histocompatibility complex molecules, thus introducing MHC-expressing DFTD cells could be a potential treatment for DFTD [1].

## Conclusions

Previous studies examining the genetic basis of disease resistance focus on a limited number and type of genes (e.g., MHC and TLR genes, [69, 70]). Here we provide evidence that a framework to examine several hundred genes involved in the immune response is better able to identify important genetic variants that confer resistance to disease. This framework can be adopted as an initial step to find solutions for a wide-range of species now in danger of extinction from disease.

## Additional files

Additional file 1: Figure S1. Coverage plots for first eight samples showing number of sequencing reads at or above specified proportions. A value at 50 Depth and 0.5 fraction means 50% of bases were at or above 50X coverage. (TIFF 149 kb)

Additional file 2: Figure S2. Coverage plots for last eight samples showing number of sequencing reads at or above specified proportions. (TIFF 124 kb)

Additional file 3: Table S1. Genes with regions deviating from neutrality. (DOCX 17 kb)

Additional file 4: Table S2. Description of *Gopherus polyphemus* samples by clinical status and diploid genotypes at several bases along the *A. superbus* venom factor 1-like and TNFRSF5-like genes for NCBI contigs NC_024232.1 and NC_024234.1 respectively. Non-clin for tortoises that were never observed with nasal discharge during the duration of the field study and Clin for tortoises that had at least one incident of mild to severe nasal discharge. CF for Cecil Field, FC for Fort Cooper, and OLD for Oldenburg. (DOCX 17 kb)

## Abbreviations

5′: Five prime
AR: Allelic richness
bp: Base pair
CF: Cecil Field/Branan Field Wildlife and Environmental Area
Clin: Clinical
DFTD: Devil facial tumor disease
DNA: Deoxyribonucleic acid
FC: Fort Cooper State Park
*F*_*ST*_: Fixation index for population subdivision
GWAS: Genome-wide association study
HE: Expected heterozygosity
HIV: Human immunodeficiency virus
HO: Observed heterozygosity
Indels: Insertions/deletions
INF-γ: Interferon gamma
km: Kilometers
MHC: Major histocompatibility complex
NCBI: National Center for Biotechnology Information
NK: Natural killer cells
Non-clin: Non-clinical
non-syn: Non-synonymous
OLD: Perry Oldenburg Wildlife and Environmental Area
PCA: Principle component analysis
PPP-RNA: Triphosphate ribonucleic acid
RADseq: Restriction-site associated DNA sequencing
RNA: Ribonucleic acid
SNPs: Single nucleotide polymorphisms
syn: Synonymous
TLR: Toll-like receptors
URTD: Upper respiratory tract disease
