# Supplementary figures and images for: Identifying genome-wide immune gene variation underlying infectious disease in wildlife populations – a next generation sequencing approach in the gopher tortoise
Source: BMC Genomics. 2018 Jan 19;19:64. doi: 10.1186/s12864-018-4452-0 (PMC5775545; doi:10.1186/s12864-018-4452-0)

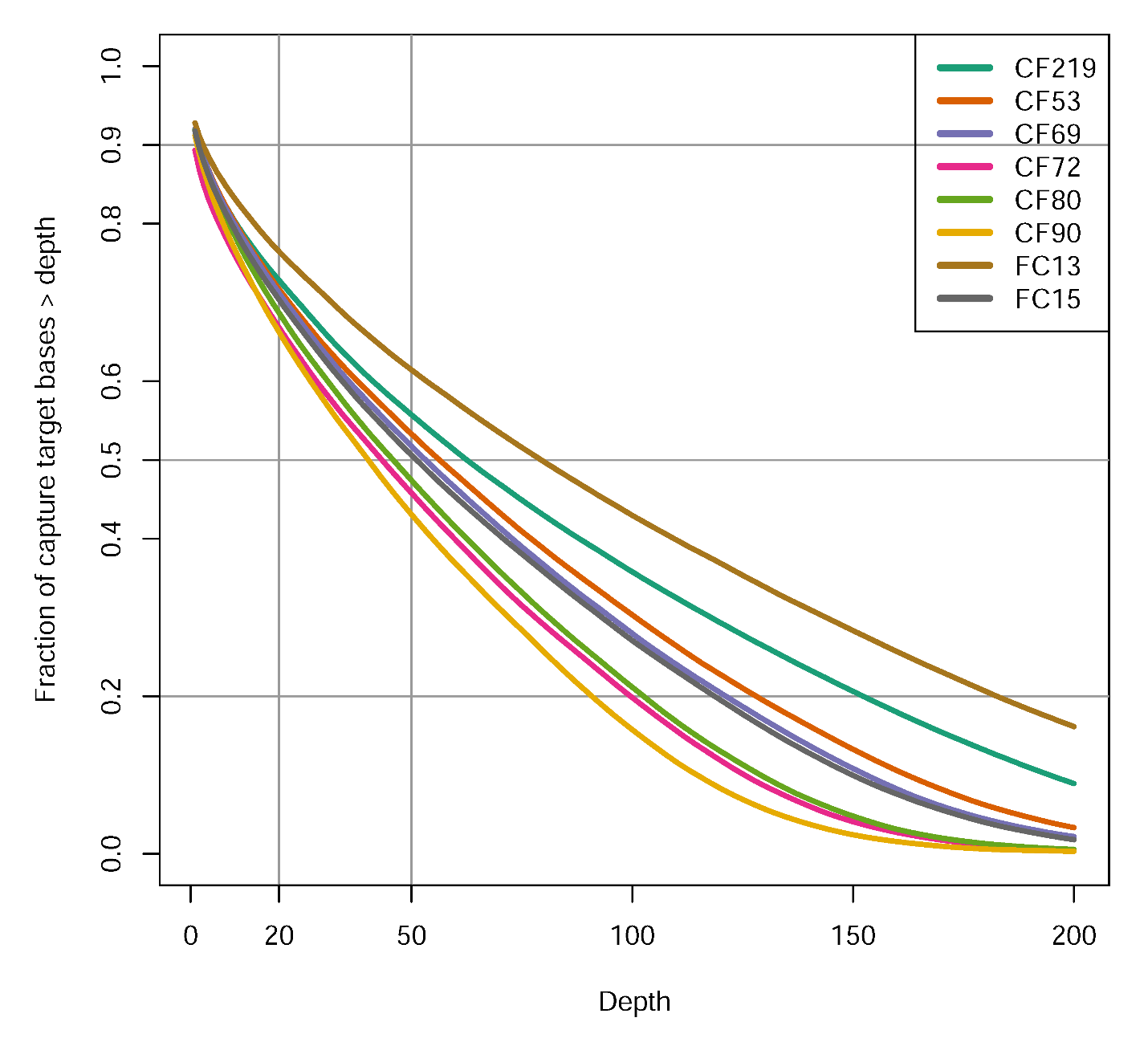

Supplement: Supplementary file 1 — Coverage plots for first eight samples showing number of sequencing reads at or above specified proportions. A value at 50 Depth and 0.5 fraction means 50% of bases were at or above 50X coverage. (TIFF 149 kb) [file 12864_2018_4452_MOESM1_ESM.tif]

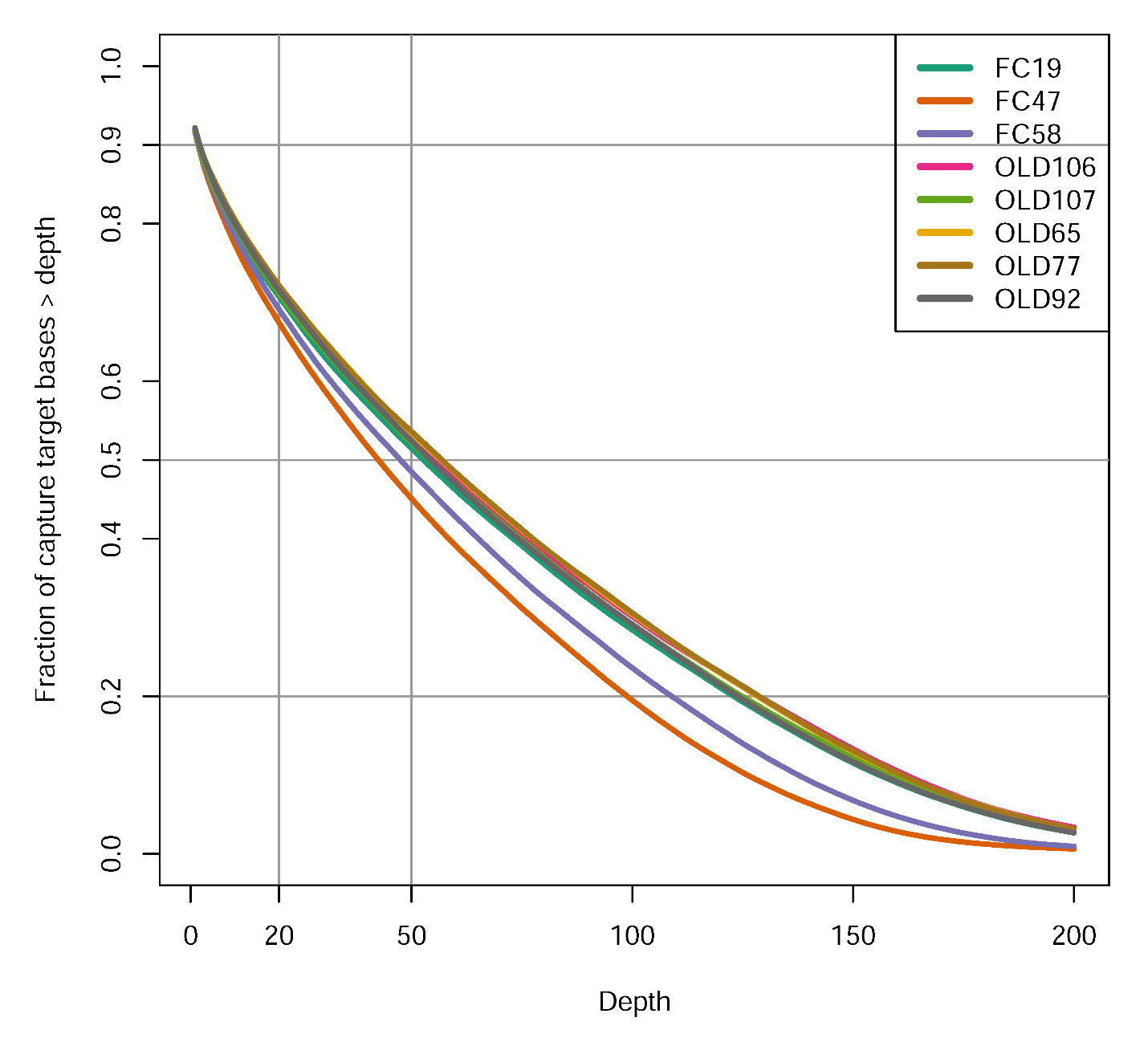

Supplement: Supplementary file 2 — Coverage plots for last eight samples showing number of sequencing reads at or above specified proportions. (TIFF 124 kb) [file 12864_2018_4452_MOESM2_ESM.tif]
