# Supplementary material for: Identifying genome-wide immune gene variation underlying infectious disease in wildlife populations – a next generation sequencing approach in the gopher tortoise
Source: BMC Genomics. 2018 Jan 19;19:64. doi: 10.1186/s12864-018-4452-0 (PMC5775545; doi:10.1186/s12864-018-4452-0)
Supplement: Supplementary file 3 — Genes with regions deviating from neutrality. (DOCX 17 kb) [file 12864_2018_4452_MOESM3_ESM.docx]

**Additional file 3: Table S1** Genes with regions deviating from neutrality.

| Gene | Function* |
| --- | --- |
| Contained region(s) with positive Tajima's *D* | |
| AQP4 | plays a role in survival of damaged and injured proliferating cells |
| BCAP29 | may be involved in transport of proteins from endoplasmic reticulum to Golgi complex |
| C1R | along with other proteins forms first part of classical pathway of complement system |
| C4BPA-like | regulates the classical pathway of complement system |
| CFH | plays an essential role in regulation of complement system activation |
| FCN2-like | may operate in a postreplication repair or a cell cycle checkpoint function |
| IFI44-like | product forms an aggregate that creates microtubular structures |
| KLRF1 | involved in the regulation of Natural Killer cell function |
| OVOS-like | has serine-type endopeptidase inhibitor activity |
| SBNO1 | Strawberry Notch Homolog 1 |
| SEC61A1 | may play a role in inserting secretory and membrane proteins into the endoplasmic reticulum |
| UL | uncharacterized locus LOC101949947 |
| ZP3-like | essential for sperm binding and zona matrix formation |
| Contained region(s) with negative Tajima's *D* | |
| BMPR1A | transmembrane serine/threonine kinases |
| DAPK1 | plays role in cell death and apoptosis |
| CD247 | plays important role in assembly of T cell receptors |
| CDC37L1 | complexes with Heat Shock Protein 90 (HSP90), which regulates protein folding |
| LCK | an important signaling molecule in selection and maturation of developing T cells |
| LRRC70 | makes cells highly sensitive to activation by cytokines and lipopolysaccharide |
| LRRFIP2 | may be involved with Toll-like receptor signaling |
| OIT3 | may be involved in hepatocellular function and development |
| OTULIN | acts as a regulator of new blood vessel development and innate immune response |
| PLEKHA1 | may be involved in the formation of signaling complexes in the plasma membrane |
| RAB17 | plays an important role in regulation of membrane traficking |
| RIPK2 | plays an essential role in modulating both the innate and adaptive branches of immune system |
| RPS6KA3 | implicated in controlling cell growth and differentiation |
| TMEM125 | Transmembrane Protein 125 |
| TRIM27-like | inhibits helper T-cell activation |
| TRIM56-like | plays a key role in innate immunity |
| VAV3 | acts as guanine nucleotide exchange factors and plays important role in new blood vessel devel. |
| ZBP1 | plays a role in innate immune response by binding to foreign DNA |
| ZNF271-like | zinc finger protein 271 |
| Contained region(s) with positive and negative Tajima's *D* | |
| IPO11 | mediates nucleocytoplasmic transport of proteins and RNAs |
| SIN3A | acts as a transcriptional repressor |
| TRAF6 | may play a role in dendritic cell maturation and activation |

*Gene functions adapted from http://www.genecards.org
