# Supplementary material for: Identifying genome-wide immune gene variation underlying infectious disease in wildlife populations – a next generation sequencing approach in the gopher tortoise
Source: BMC Genomics. 2018 Jan 19;19:64. doi: 10.1186/s12864-018-4452-0 (PMC5775545; doi:10.1186/s12864-018-4452-0)
Supplement: Supplementary file 4 — Description of Gopherus polyphemus samples by clinical status and diploid genotypes at several bases along the A. superbus venom factor 1-like and TNFRSF5-like genes for NCBI contigs NC_024232.1 and NC_024234.1 respectively. Non-clin for tortoises that were never observed with nasal discharge during the duration of the field study and Clin for tortoises that had at least one incident of mild to severe nasal discharge. CF for Cecil Field, FC for Fort Cooper, and OLD for Oldenburg. (DOCX 17 kb) [file 12864_2018_4452_MOESM4_ESM.docx]

**Additional file 4: Table S2** Description of *Gopherus polyphemus* samples by clinical status and diploid genotypes at several bases along the *A. superbus* venom factor 1-like and TNFRSF5-like genes for NCBI contigs NC_024232.1 and NC_024234.1 respectively. Non-clin for tortoises that were never observed with nasal discharge during the duration of the field study and Clin for tortoises that had at least one incident of mild to severe nasal discharge. CF for Cecil Field, FC for Fort Cooper, and OLD for Oldenburg.

| Sample | Clinical status | NC_024232.1 Bases | | | NC_024234.1 Bases |  |
| --- | --- | --- | --- | --- | --- | --- |
|  |  | 1105284 | 1105940 | 1105959 | 2112869 | |
| FC19 | Non-clin | G,G | A,A | A,A | GCA,GCA | |
| OLD77 | Non-clin | G,G | A,A | A,A | GCA,GCA | |
| CF53 | Non-clin | G,G | A,A | A,A | GCA,GCA | |
| CF69 | Non-clin | G,G | A,A | A,A | GCA,G | |
| FC58 | Non-clin | C,G | G,A | G,A | G,G | |
| OLD106 | Non-clin | C,G | G,A | G,A | G,G | |
| CF72 | Clin | C,C | G,G | G,G | G,G | |
| CF80 | Clin | C,C | G,G | G,G | G,G | |
| CF90 | Clin | C,C | G,G | G,G | G,G | |
| CF219 | Clin | C,C | G,G | G,G | G,G | |
| FC13 | Clin | C,G | G,A | G,A | G,G | |
| FC15 | Clin | C,C | G,G | G,G | G,G | |
| FC47 | Clin | C,C | G,G | G,G | G,G | |
| OLD65 | Clin | C,G | G,A | G,A | G,G | |
| OLD92 | Clin | C,C | G,G | G,G | GCA,G | |
| OLD107 | Clin | C,G | G,A | G,A | G,G | |
